# Influenza Morbidity and Mortality in Elderly Patients Receiving Statins: A Cohort Study

**DOI:** 10.1371/journal.pone.0008087

**Published:** 2009-11-30

**Authors:** Jeffrey C. Kwong, Ping Li, Donald A. Redelmeier

**Affiliations:** 1 The Institute for Clinical Evaluative Sciences, Toronto, Ontario, Canada; 2 Departments of Family and Community Medicine, University of Toronto, Toronto, Ontario, Canada; 3 Departments of Medicine and Health Policy, Management, and Evaluation, University of Toronto, Toronto, Ontario, Canada; 4 The Dalla Lana School of Public Health, University of Toronto, Toronto, Ontario, Canada; CIET, Canada

## Abstract

**Background:**

Statins possess immunomodulatory properties and have been proposed for reducing morbidity during an influenza pandemic. We sought to evaluate the effect of statins on hospitalizations and deaths related to seasonal influenza outbreaks.

**Methodology/Principal Findings:**

We conducted a population-based cohort study over 10 influenza seasons (1996 to 2006) using linked administrative databases in Ontario, Canada. We identified all adults older than 65 years who had received an influenza vaccination prior to the start of influenza season and distinguished those also prescribed statins (23%) from those not also prescribed statins (77%). Propensity-based matching, which accounted for each individual's likelihood of receiving a statin, yielded a final cohort of 2,240,638 patients, exactly half of whom received statins. Statins were associated with small protective effects against pneumonia hospitalization (odds ratio [OR] 0.92; 95% CI 0.89–0.95), 30-day pneumonia mortality (0.84; 95% CI 0.77–0.91), and all-cause mortality (0.87; 95% CI 0.84–0.89). These protective effects attenuated substantially after multivariate adjustment and when we excluded multiple observations for each individual, declined over time, differed across propensity score quintiles and risk groups, and were unchanged during post-influenza season periods. The main limitations of this study were the observational study design, the non-specific outcomes, and the lack of information on medications while hospitalized.

**Conclusions/Significance:**

Statin use is associated with a statistically significant but minimal protective effect against influenza morbidity that can easily be attributed to residual confounding. Public health officials and clinicians should focus on other measures to reduce morbidity and mortality from the next influenza pandemic.

## Introduction

Influenza causes an estimated 1 million worldwide deaths annually[1] and a future influenza pandemic as severe as the 1918-20 pandemic would cause about 62 million deaths.[2] Some strains of avian influenza have a case fatality rate of around 60%.[3] The ultimate impact of the current influenza A(H1N1) pandemic remains uncertain. Availability of a vaccine against the pandemic strain is largely restricted to a select group of countries with manufacturers. The utility of non-vaccine antiviral medications is similarly hampered by limited production capacity and viral resistance. The strategy of public health measures (e.g., social distancing and face masks) is of uncertain effectiveness.

Recommendations for inexpensive generic medications that target the host immune response to mitigate the effects of influenza are a topic of broad interest.[4] Several studies have demonstrated excessive elevations in pro-inflammatory cytokines and chemokines associated with severe influenza infections.[5]–[8] Statins, therefore, have real potential due to their ability to reduce levels of pro-inflammatory cytokines and increase levels of anti-inflammatory cytokines.[9]–[17] Treatment with statins has been shown in some non-randomized studies to be associated with decreased progression to severe sepsis and reduced mortality from sepsis.[18]–[20] Previous studies examining the benefits of statins on pneumonia morbidity and mortality, however, have found conflicting results with some showing large protective effects[21]–[26] and others finding no protection.[27], [28]

The prospect of using statins to combat an influenza pandemic is enticing because they are widely available, have a long shelf-life, and will not induce viral resistance. Clinicians also have extensive familiarity with their pharmacologic profile. However, if statins are actually ineffective their widespread use could potentially cause more harm than good due to adverse drug reactions such as hepatitis and myositis. Therefore, accurately estimating the effectiveness of statins in preventing serious influenza-related outcomes is a priority for global public health. The purpose of this study was to evaluate the effect of statins on hospitalizations and deaths related to influenza.

## Methods

### Setting and Patients

We assembled a retrospective cohort of patients by linking multiple administrative health-care databases over a ten year period (1996 to 2006) in the province of Ontario, Canada's most populous province. The population included 12.2 million as of 2006, including 1.6 million people aged 65 or older. All elderly patients had free access to hospital care, physician services, and prescription medications. Ethics approval was obtained from the Sunnybrook Health Sciences Centre Research Ethics Board, Toronto, Canada.

The study period included ten influenza seasons (1996–97 to 2005–06). We used respiratory virus surveillance data from a network of sentinel laboratories to identify influenza season periods, in accord with previous research.[29] We identified the start and end of influenza seasons as the first and last occurrences of at least two consecutive weeks during which the weekly percentage of tests positive for influenza was 5% or greater. We chose this more inclusive definition for influenza season to increase event counts (different intervals tested in sensitivity analyses).

We used several large, validated databases for this study. The Ontario Drug Benefits (ODB) database recorded all prescription medications dispensed to elderly patients.[30], [31] The Canadian Institute for Health Information (CIHI) Discharge Abstract database recorded detailed information about diagnoses and procedures for all hospitalizations.[32] The Ontario Health Insurance Plan (OHIP) database recorded physician billing claims for inpatient and outpatient services for 98% of Ontario physicians.[33] The Ontario Registered Persons Database (RPDB) recorded the vital status of all individuals in the province.[34] Encrypted unique identifiers allowed for linkage of individuals across all databases.

We included all patients older than 65 years who received an influenza vaccination, measured using physician billing claims at least 2 weeks prior to the start of an influenza season in OHIP (G538/G539 during October and November or G590/G591 at any time).[35] These codes have a specificity of 90% and a positive predictive value of 96% for influenza vaccination. [35] The small number of false positive codes reflects individuals who may have received non-influenza vaccines. The study period included ten separate influenza seasons; hence, each patient could be included in the cohort a maximum of ten times (different models tested in sensitivity analyses).

Patients were characterized as statin users if they received one or more prescriptions for a statin during the 90 days preceding the start of an influenza season (index date). The six statins available during the study period were atorvastatin, cerivastatin, fluvastatin, lovastatin, pravastatin, and simvastatin. Individuals who did not receive a statin prescription during this 3-month period were characterized as controls.

The primary study outcome was hospitalization for pneumonia (ICD9-CM codes 480–487 and ICD10-CA codes J10-J18) during influenza season. Secondary outcomes were deaths from any cause within 30 days following such an admission and deaths from all causes during influenza season.

We obtained individual data on more than 100 demographic factors, previous health care use, specific procedures, current prescription medications, and risk factors for influenza (Table S1) from multiple datasets.[36], [37] Risk factors for influenza were defined as listed in the Canadian Immunization Guide indications.[38] Comorbidities were defined based on an adaptation of the adjusted clinical group as any mention of the diagnosis in the outpatient or hospitalization datasets during the prior three years[39].

For each statin user, we identified one control who did not receive a statin using propensity-based matching.[40] To do so, we first calculated propensity scores for statin treatment in each patient-year in the cohort based on an extensive list of factors potentially related to statin use or influenza (Table S1).[38] We next matched each statin user to a smaller pool of controls by sex, age (plus or minus one year), and influenza season. Ultimately, we selected in a 1∶1 fashion the control with the closest propensity score (within 0.2 SD) to each statin user. We discarded any unmatched statin users and unmatched controls.

### Statistical Analyses

We used conditional logistic regression models to estimate the risk of an outcome during influenza season periods in statin users compared to controls for the entire cohort and for each influenza season separately. We also performed adjusted analyses that controlled for the following pre-specified covariates: age, sex, chronic institutionalization, number of hospitalizations in the prior three years, number of medications prescribed in the prior year, and risk factors for influenza-related complications (ischemic heart disease, congestive heart failure, chronic obstructive pulmonary disease, diabetes mellitus with complications, serious cancers, chronic renal failure, and dementia and delirium).

### Sensitivity Analyses

We conducted multiple sensitivity analyses to test the robustness of our findings. We repeated the analysis without propensity score matching and instead used standard multivariate regression methods. We also repeated the analysis using a more restrictive definition for influenza season periods that included only the five consecutive weeks of greatest influenza activity.[29] To eliminate potential bias arising from including individuals repeatedly over multiple influenza seasons, we performed an analysis that included only the first appearance of each individual. To check the effect of statins on outcomes when influenza was not circulating in the community, we also repeated the analyses for the post-influenza season (defined as June 1 to September 30 each year), using the 90 days prior to the start of the post-influenza season to characterize statin use. To assess the specificity of the association between statin use and the main study outcomes, we replicated the analysis using negative tracer outcomes for which no associations were expected: hospitalizations for pericarditis (ICD-9-CM codes 391.0, 393, 420, 423.1-423.2 and ICD-10-CA codes I01.0, I09.2, I30, I31.0-I31.1, I32) and for motor vehicle collisions (ICD-9-CM codes E810-E825 and ICD-10-CA codes V01-V79).

We conducted several subgroup analyses by dividing the cohort based on: sex; age (less than versus greater than or equal to the median age); hospitalizations in the past three years (none versus any); the number of current medications prescribed (fewer than versus greater than or equal to nine); the presence of any of the influenza-related risk factors listed in Table 1 (none versus any); and the type of statin prescribed (atorvastatin, simvastatin, pravastatin, and other statins). To examine the effect of statins across likelihood of receiving statins, we divided the cohort into quintiles based on the propensity score and repeated the analysis for each outcome.

**Table 1:** Baseline characteristics in the matched cohort.

	Statin group	Control group
	(n = 1,120,319)	(n = 1,120,319)
**Demographic factors**		
Age (mean - years) (SD)	74.34 (5.78)	74.34 (5.78)
Sex (male)	505,264 (45.1%)	505,264 (45.1%)
Institutional care status	11,614 (1.0%)	9,971 (0.9%)
**Health-care use**		
Number of admissions (past 3 years)	0.58 (1.19)	0.57 (1.16)
Number of medications (past year)	9.91 (5.72)	9.94 (6.73)
**Risk factors for influenza**		
Ischemic heart disease + AMI	465,573 (41.6%)	462,182 (41.3%)
Congestive heart failure	122,505 (10.9%)	120,252 (10.7%)
Emphysema, chronic bronchitis, COPD	129,026 (11.5%)	128,667 (11.5%)
DM with complications	22,940 (2.0%)	22,915 (2.0%)
Serious cancers	40,295 (3.6%)	40,622 (3.6%)
Chronic renal failure	39,851 (3.6%)	39,304 (3.5%)
Dementia and delirium	49,574 (4.4%)	48,216 (4.3%)

AMI = acute myocardial infarction. COPD = chronic obstructive pulmonary disease.

Analyses were performed using SAS 9.1 (SAS Institute Inc., Cary, NC). All tests were two-tailed and we used p<0.05 as the level of statistical significance.

## Results

**Figure 1:**
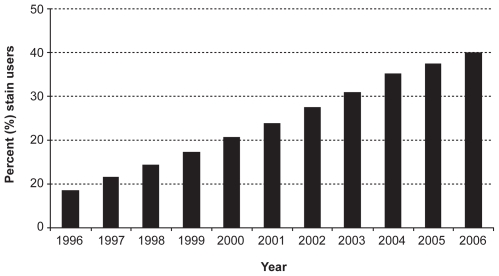
Prevalence of statin use. Percentage of elderly population who received at least one prescription for a statin each year over the study period.

The percentage of elderly individuals in Ontario who received at least one prescription for a statin during a given year increased steadily over the study period, from 8.6% in 1996 to 40.0% in 2006 (Figure 1). We identified 1,565,074 statin users and 5,112,221 controls over the ten year study interval. Characteristics of the cohort before and after matching are presented in Table S1. After matching, 1,120,319 pairs of statin users and non-users remained in the cohort. The two matched groups appeared very similar (Table 1). There were 13,027 pneumonia hospitalizations, 2,205 deaths within 30 days of such an admission, and 17,472 deaths from all causes over the ten influenza seasons.

**Figure 2:**
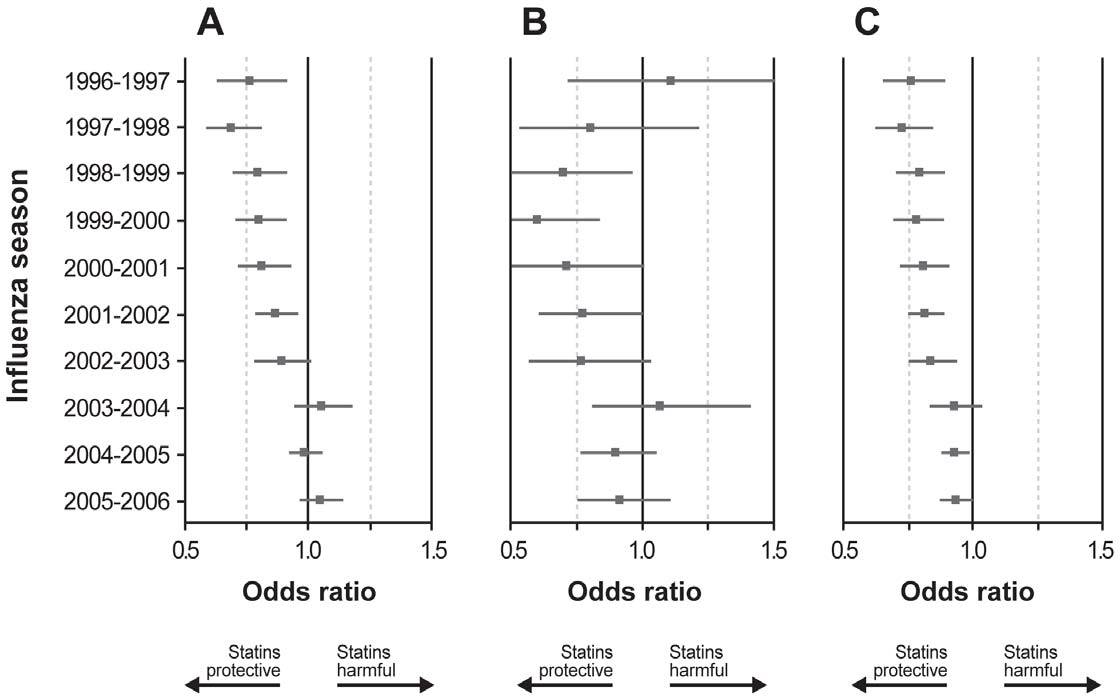
Year-specific analyses. Odd ratios for statin use and pneumonia hospitalization (A), 30-day pneumonia mortality (B), and all-cause mortality (C), by influenza season, with patients not treated with statins as the reference group. Horizontal lines show 95% CI.

In the primary analysis, statin use showed a small but statistically significant apparent protective association against hospitalization (OR = 0.92; 95% CI 0.89–0.95) (Table 2). This apparent effect was reduced substantially in the adjusted analysis (0.97; 95% CI 0.94–1.00). When examining the effect by influenza season, statins appeared to be protective during six seasons (1996–97 to 2001–02) but not during four seasons (2002–03 to 2005–06) (Figure 2).

**Table 2:** Statins and influenza morbidity.

	Odds ratio (95% CI)		
	Pneumonia hospitalization	30-day pneumonia mortality	All-cause mortality
With propensity score matching – crude	0.92 (0.89–0.95)	0.84 (0.77–0.91)	0.87 (0.84–0.89)
With propensity score matching – adjusted	0.97 (0.94–1.00)	0.90 (0.82–0.98)	0.91 (0.88–0.94)
Without propensity score matching – crude	0.72 (0.71–0.74)	0.50 (0.47–0.53)	0.56 (0.55–0.57)
Without propensity score matching – adjusted	0.96 (0.93–0.99)	0.89 (0.83–0.95)	0.90 (0.88–0.92)
Restrict to peak influenza weeks – crude	0.89 (0.85–0.94)	0.79 (0.70–0.91)	0.86 (0.82–0.90)
Restrict to peak influenza weeks – adjusted	0.95 (0.90–1.00)	0.86 (0.75–0.98)	0.91 (0.86–0.95)
Limit to first appearance of individual – crude	0.94 (0.89–0.99)	0.97 (0.84–1.11)	0.92 (0.88–0.97)
Limit to first appearance of individual - adjusted	0.98 (0.93–1.04)	1.01 (0.88–1.17)	0.95 (0.90–1.00)
Change to post-influenza season – crude	0.93 (0.90–0.96)	0.81 (0.74–0.89)	0.79 (0.76–0.81)
Change to post-influenza season – adjusted	0.97 (0.93–1.00)	0.85 (0.78–0.92)	0.80 (0.78–0.82)
By propensity score quintile			
Quintile 1 (least likely to be on a statin)	1.00 (0.93–1.07)	0.94 (0.80–1.09)	0.99 (0.94–1.05)
Quintile 2	0.86 (0.80–0.93)	0.73 (0.60–0.89)	0.86 (0.81–0.92)
Quintile 3	0.90 (0.83–0.97)	0.84 (0.69–1.02)	0.83 (0.78–0.89)
Quintile 4	0.90 (0.83–0.98)	0.88 (0.72–1.09)	0.81 (0.76–0.87)
Quintile 5 (most likely to be on a statin)	0.92 (0.85–0.99)	0.78 (0.64–0.96)	0.81 (0.75–0.87)

The apparent protective association with statins was slightly larger against 30-day pneumonia mortality (0.84; 95% CI 0.77–0.91) and all-cause mortality (0.87; 95% CI 0.84–0.89) (Table 2). These estimates also shifted towards the null after multivariate adjustment. For all-cause mortality but not for 30-day pneumonia mortality, the year-specific analyses displayed similar temporal inconsistencies as observed for hospitalizations (Figure 2).

Without propensity score matching, statin use was associated with apparent protection against all three outcomes in the crude analysis that attenuated toward the null with adjustment (Table 2). Using a more restrictive definition of influenza season yielded similar results. Limiting the analysis to include only the first appearance of an individual during the study period also shifted the estimates towards the null, particularly for 30-day pneumonia mortality. Associations calculated for the post-influenza season analyses were either similar to or more pronounced than those during influenza peaks. As anticipated, no association was detected between statin use and hospitalizations for pericarditis (1.05; 95% CI 0.78–1.42) or motor vehicle collisions (1.00; 95% CI 0.83–1.21).

**Figure 3:**
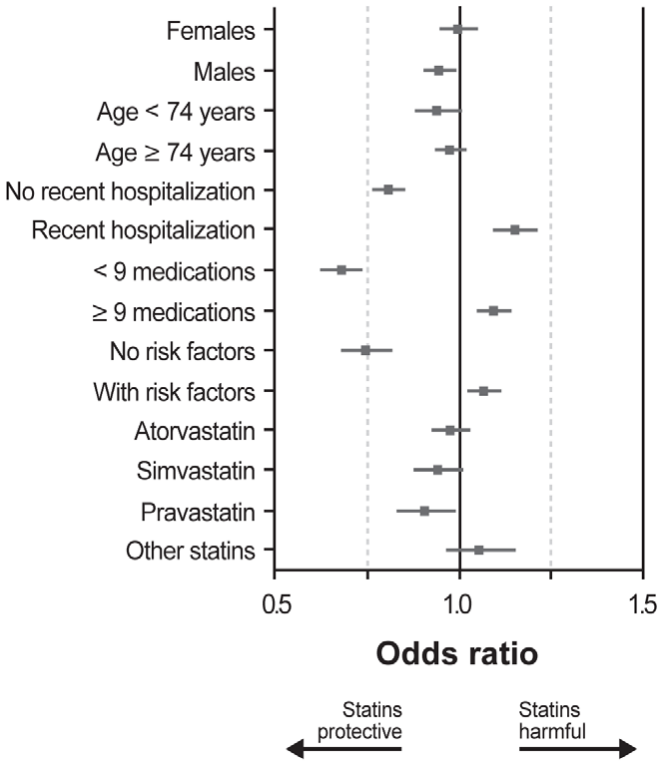
Subgroup analyses. Odd ratios for statin use and pneumonia hospitalization, with patients not treated with statins as the reference group. Horizontal lines show 95% CI.

In the subgroup analyses, women obtained no protection from statin use, whereas men showed a small trend (Figure 3). Differences between older and younger individuals were minimal. In contrast, dividing the cohort by recent hospitalizations, number of prescription medications, and presence of risk factors for influenza dramatically influenced the effect of statins, with statins appearing protective for healthier individuals and harmful for sicker individuals. When the analyses were repeated with the cohort divided into quintiles based on the propensity score, individuals in quintile 1 (i.e., those least likely to receive statins) experienced no protection from statins against any outcome (1.00; 95% CI 0.93–1.07 for pneumonia hospitalization; 0.94; 95% CI 0.80–1.09 for 30-day pneumonia mortality; and 0.99; 95% CI 0.94–1.05 for all-cause mortality) (Table 2).

## Discussion

We found a small, statistically significant apparent protective association of statins against influenza morbidity and mortality. However the association was inconsistent and could be easily attributed to hidden confounding. The potential protective association was not consistent over the study period or across subgroups based on health status, propensity score, or when we excluded multiple observations for each individual. Moreover, the finding was not distinct to outcomes observed during influenza seasons, although it is possible that any protective effect of statins, if present, might not be specific to pneumonia resulting from influenza infection. Together, these findings cast doubt on the role of statins for treating an influenza pandemic.

The protection estimated in this study is much smaller than in previous reports. For instance, van de Garde *et al*. and Schlienger *et al*. reported 50% and 37% protection against pneumonia hospitalization, respectively.[22], [23] The comparable estimate from our analysis is 8% (3% after adjustment). For 30-day pneumonia mortality, previous estimates ranged from 31% to 64%, whereas our study showed 16% protection (10% after adjustment).[21], [23]–[26] The reduced benefits may indicate better control of confounding due to more finely stratified disease groupings. The use of such a small number of variables in those studies was likely insufficient to account for most of the differences between statin users and non-users.

Despite extensive statistical modeling, our study suggests residual confounding, sometimes referred to as the “healthy user” effect, persists. Some patients who tend to engage in health-seeking behaviours are more adherent to medical advice and are sometimes ironically misclassified in administrative databases as having greater numbers of coexisting conditions as other patients.[27], [28], [41] The presence of the healthy user effect is congruent with the change in protection from statins observed over time in this study (since many new therapies are initially more likely to be adopted by healthy users, and then followed by gradual dissemination to sicker individuals).[42] Therefore, the spurious benefits of statins will appear to decline over time. Further evidence is the lack of benefit observed in the analysis that included individuals only once. Healthy users tend to seek influenza vaccinations regularly and therefore were likely to be included in the analysis repeatedly; their presence in multiple years would bias the estimates of protection.

This study had several limitations that merit emphasis. First, this was an observational study, and we may not have been able to entirely eliminate the effect of unmeasured confounders. Previous work has demonstrated that propensity score methods are unable to fully control for residual confounding.[43] Second, our outcomes are non-specific and may have been due to causes other than influenza. Third, we did not have information on medications while hospitalized, although this was relevant only for mortality outcomes. Finally, we used health administrative data for assessing covariates with questionable accuracy of diagnostic codes.

Among the strengths of this study, the most notable is its size. With over 2.2 million person-years of observation resulting in 13,027 pneumonia hospitalizations during ten influenza seasons of varying severity, our data form the largest study to date by a factor of 75. We eliminated non-compliance with vaccination as a confounder and examined actual treatment with statins in a population-based community setting. Another strength is that we used a large number of variables to develop the propensity score model, with fine stratification of disease groupings. Lastly, we used laboratory surveillance data to define influenza season periods.

Despite the exciting prospect suggested by previous studies, our data suggest that statins do not substantially reduce morbidity and mortality from influenza. Public health officials and clinicians should focus on other measures to reduce morbidity and mortality from the next influenza pandemic.

## Supporting Information

Table S1 Cohort characteristics before and after matching.
(0.22 MB DOC)
