# Supplementary material for: Influenza Morbidity and Mortality in Elderly Patients Receiving Statins: A Cohort Study
Source: PLoS One. 2009 Nov 30;4(11):e8087. doi: 10.1371/journal.pone.0008087 (PMC2778952; doi:10.1371/journal.pone.0008087)
Supplement: Table S1 — Cohort characteristics before and after matching. (0.22 MB DOC) [file pone.0008087.s001.doc]

**Table S**1: Cohort characteristics before and after matching

|  | **Before match** | | **After match** | |
| --- | --- | --- | --- | --- |
|  | Statins | Control | Statins | Control |
|  | N=1,565,074 | N=5,112,211 | N=1,120,319 | N=1,120,319 |
| **Demographic factors** |  |  |  |  |
| Age (years) |  |  |  |  |
| Mean (SD) | 74.20 (5.67) | 75.83 (6.91) | 74.34 (5.78) | 74.34 (5.78) |
| Median (interquartile range) | 73 (70-78) | 75 (70-80) | 74 (70-78) | 74 (70-78) |
| Sex (male) | 747,226 (47.7%) | 2,113,571 (41.3%) | 505,264 (45.1%) | 505,264 (45.1%) |
| Income quintiles |  |  |  |  |
| 1 | 293,802 (18.8%) | 952,369 (18.6%) | 215,152 (19.2%) | 215,216 (19.2%) |
| 2 | 335,976 (21.5%) | 1,077,105 (21.1%) | 245,188 (21.9%) | 245,237 (21.9%) |
| 3 | 313,595 (20.0%) | 1,004,588 (19.7%) | 230,032 (20.5%) | 230,452 (20.6%) |
| 4 | 290,574 (18.6%) | 932,904 (18.2%) | 212,588 (19.0%) | 212,774 (19.0%) |
| 5 | 293,480 (18.8%) | 1,011,560 (19.8%) | 217,359 (19.4%) | 216,640 (19.3%) |
| Missing | 37,647 (2.4%) | 133,685 (2.6%) |  |  |
| Institutional care status | 15,000 (1.0%) | 179,915 (3.5%) | 11,614 (1.0%) | 9,971 (0.9%) |
| Rural residence |  |  |  |  |
| Yes | 198,021 (12.7%) | 756,101 (14.8%) | 141,618 (12.6%) | 141,029 (12.6%) |
| No | 1,365,428 (87.2%) | 4,349,282 (85.1%) | 978,701 (87.4%) | 979,290 (87.4%) |
| Missing | 1,625 (0.1%) | 6,828 (0.1%) |  |  |
| Influenza season |  |  |  |  |
| 1996-1997 | 40,722 (2.6%) | 444,339 (8.7%) | 36,598 (3.3%) | 36,598 (3.3%) |
| 1997-1998 | 66,969 (4.3%) | 502,082 (9.8%) | 55,535 (5.0%) | 55,535 (5.0%) |
| 1998-1999 | 79,231 (5.1%) | 485,373 (9.5%) | 62,720 (5.6%) | 62,720 (5.6%) |
| 1999-2000 | 93,737 (6.0%) | 468,530 (9.2%) | 72,801 (6.5%) | 72,801 (6.5%) |
| 2000-2001 | 147,375 (9.4%) | 606,767 (11.9%) | 112,893 (10.1%) | 112,893 (10.1%) |
| 2001-2002 | 184,895 (11.8%) | 619,045 (12.1%) | 136,188 (12.2%) | 136,188 (12.2%) |
| 2002-2003 | 199,854 (12.8%) | 533,903 (10.4%) | 140,623 (12.6%) | 140,623 (12.6%) |
| 2003-2004 | 159,990 (10.2%) | 354,169 (6.9%) | 108,347 (9.7%) | 108,347 (9.7%) |
| 2004-2005 | 285,050 (18.2%) | 542,570 (10.6%) | 185,741 (16.6%) | 185,741 (16.6%) |
| 2005-2006 | 307,251 (19.6%) | 555,433 (10.9%) | 208,873 (18.6%) | 208,873 (18.6%) |
|  |  |  |  |  |
| **Health-care use** |  |  |  |  |
| Outpatient clinic visit (days) | 20.02 (15.30) | 17.99 (16.04) | 19.14 (15.29) | 19.21 (14.33) |
| Cardiologist visit (days) | 0.94 (2.72) | 0.37 (1.60) | 0.73 (2.31) | 0.69 (2.31) |
| Neurologist visit (days) | 0.14 (0.90) | 0.12 (0.83) | 0.13 (0.88) | 0.14 (0.81) |
| Psychiatrist visit (days) | 0.13 (1.66) | 0.16 (1.98) | 0.13 (1.71) | 0.13 (1.53) |
| Optometrist visit (days) | 0.53 (0.83) | 0.47 (0.80) | 0.52 (0.82) | 0.52 (0.84) |
| Ophthalmologist visit (days) | 0.96 (2.00) | 0.91 (1.96) | 0.95 (1.99) | 0.96 (1.99) |
| Cholesterol test (yes/no) | 1,234,743 (78.9%) | 2,094,574 (41.0%) | 825,218 (73.7%) | 854,782 (76.3%) |
| Number of cholesterol tests (past year) | 4.67 (4.12) | 1.55 (2.45) | 3.79 (3.46) | 3.66 (3.46) |
| Number of admissions (past 3 years) | 0.65 (1.26) | 0.55 (1.15) | 0.58 (1.19) | 0.57 (1.16) |
| Number of medications (past year) | 10.63 (6.15) | 7.68 (5.84) | 9.91 (5.72) | 9.94 (6.73) |
| Home care receipt (past 3 months) | 116,043 (7.4%) | 505,378 (9.9%) | 85,043 (7.6%) | 83,384 (7.4%) |
|  |  |  |  |  |
| **Procedures** |  |  |  |  |
| Coronary angiography | 188,715 (12.1%) | 189,386 (3.7%) | 99,575 (8.9%) | 93,017 (8.3%) |
| Coronary bypass grafting | 123,995 (7.9%) | 95,995 (1.9%) | 61,035 (5.4%) | 55,282 (4.9%) |
| Percutaneous coronary intervention | 306,418 (19.6%) | 307,406 (6.0%) | 162,297 (14.5%) | 152,445 (13.6%) |
| Echocardiography | 515,099 (32.9%) | 932,385 (18.2%) | 325,234 (29.0%) | 325,255 (29.0%) |
| Electrocardiography | 1,473,601 (94.2%) | 4,604,361 (90.1%) | 1,046,231 (93.4%) | 1,051,981 (93.9%) |
| Peripheral bypass grafting | 20,358 (1.3%) | 34,516 (0.7%) | 12,412 (1.1%) | 12,304 (1.1%) |
| Holter monitor | 434,422 (27.8%) | 1,080,991 (21.1%) | 292,486 (26.1%) | 295,990 (26.4%) |
| Stress test | 863,233 (55.2%) | 1,808,011 (35.4%) | 563,167 (50.3%) | 570,241 (50.9%) |
| Cardiac pacemaker procedures | 73,331 (4.7%) | 168,510 (3.3%) | 47,133 (4.2%) | 47,015 (4.2%) |
| Valve and septal surgery | 23,153 (1.5%) | 40,790 (0.8%) | 14,472 (1.3%) | 14,815 (1.3%) |
| Carotid endarterectomy | 24,237 (1.5%) | 25,282 (0.5%) | 12,945 (1.2%) | 12,175 (1.1%) |
| Dialysis | 8,611 (0.6%) | 14,852 (0.3%) | 5,586 (0.5%) | 5,764 (0.5%) |
| Bone densitometry | 647,193 (41.4%) | 1,931,128 (37.8%) | 473,062 (42.2%) | 481,278 (43.0%) |
|  |  |  |  |  |
| **Risk factors for influenza** |  |  |  |  |
| Cardiovascular diseases |  |  |  |  |
| Ischemic heart disease + AMI | 752,239 (48.1%) | 1,282,894 (25.1%) | 465,573 (41.6%) | 462,182 (41.3%) |
| Congenital heart disease | 10,303 (0.7%) | 21,117 (0.4%) | 6,662 (0.6%) | 6,805 (0.6%) |
| Congestive heart failure | 187,259 (12.0%) | 477,756 (9.3%) | 122,505 (10.9%) | 120,252 (10.7%) |
| Cardiac valve disorders | 46,518 (3.0%) | 102,328 (2.0%) | 29,598 (2.6%) | 29,370 (2.6%) |
| Cardiomyopathy | 9,311 (0.6%) | 15,298 (0.3%) | 5,503 (0.5%) | 5,458 (0.5%) |
| Cardiac arrhythmia | 284,516 (18.2%) | 734,154 (14.4%) | 189,999 (17.0%) | 189,859 (16.9%) |
| Generalized atherosclerosis | 89,622 (5.7%) | 161,313 (3.2%) | 54,215 (4.8%) | 52,967 (4.7%) |
| Hypertension, w/o major complications | 1,123,642 (71.8%) | 3,000,256 (58.7%) | 781,480 (69.8%) | 792,676 (70.8%) |
| Hypertension, with major complications | 56,261 (3.6%) | 117,129 (2.3%) | 36,636 (3.3%) | 36,916 (3.3%) |
| Cardiovascular disorders, other | 57,784 (3.7%) | 78,953 (1.5%) | 30,352 (2.7%) | 27,495 (2.5%) |
| Respiratory diseases |  |  |  |  |
| Acute lower respiratory tract infection | 487,432 (31.1%) | 1,579,196 (30.9%) | 345,761 (30.9%) | 347,070 (31.0%) |
| Emphysema, chronic bronchitis, COPD | 179,359 (11.5%) | 658,218 (12.9%) | 129,026 (11.5%) | 128,667 (11.5%) |
| Asthma, with and w/o status asthmaticus | 160,084 (10.2%) | 550,230 (10.8%) | 115,867 (10.3%) | 116,732 (10.4%) |
| Respiratory disorders, other | 80,859 (5.2%) | 234,818 (4.6%) | 53,510 (4.8%) | 52,710 (4.7%) |
| Pulmonary embolism | 4,955 (0.3%) | 16,015 (0.3%) | 3,389 (0.3%) | 3,370 (0.3%) |
| Respiratory failure | 13,193 (0.8%) | 32,730 (0.6%) | 8,063 (0.7%) | 7,767 (0.7%) |
| Diabetes mellitus |  |  |  |  |
| Uncomplicated diabetes mellitus | 447,443 (28.6%) | 822,204 (16.1%) | 288,876 (25.8%) | 290,677 (25.9%) |
| Diabetes mellitus with complications | 37,229 (2.4%) | 67,802 (1.3%) | 22,940 (2.0%) | 22,915 (2.0%) |
| Lipid disorders | 755,568 (48.3%) | 693,882 (13.6%) | 448,841 (40.1%) | 456,727 (40.8%) |
| Cancer |  |  |  |  |
| Serious cancers | 54,459 (3.5%) | 219,399 (4.3%) | 40,295 (3.6%) | 40,622 (3.6%) |
| Malignant neoplasms of the skin | 167,574 (10.7%) | 551,910 (10.8%) | 119,507 (10.7%) | 119,170 (10.6%) |
| Low impact malignant neoplasms | 59,408 (3.8%) | 207,409 (4.1%) | 42,762 (3.8%) | 42,947 (3.8%) |
| High impact malignant neoplasms | 35,677 (2.3%) | 147,700 (2.9%) | 26,468 (2.4%) | 26,622 (2.4%) |
| Malignant neoplasms, breast | 36,823 (2.4%) | 150,566 (2.9%) | 28,392 (2.5%) | 28,360 (2.5%) |
| Malignant neoplasms, cervix, uterus | 6,278 (0.4%) | 26,364 (0.5%) | 4,776 (0.4%) | 4,856 (0.4%) |
| Malignant neoplasms, ovary | 2,854 (0.2%) | 11,707 (0.2%) | 2,211 (0.2%) | 2,184 (0.2%) |
| Malignant neoplasms, esophagus | 1,695 (0.1%) | 7,214 (0.1%) | 1,217 (0.1%) | 1,190 (0.1%) |
| Malignant neoplasms, kidney | 2,294 (0.1%) | 7,077 (0.1%) | 1,591 (0.1%) | 1,636 (0.1%) |
| Malignant neoplasms, liver and biliary tract | 1,427 (0.1%) | 7,792 (0.2%) | 1,104 (0.1%) | 1,177 (0.1%) |
| Malignant neoplasms, lung | 14,483 (0.9%) | 55,810 (1.1%) | 10,699 (1.0%) | 10,866 (1.0%) |
| Malignant neoplasms, lymphomas | 9,227 (0.6%) | 36,080 (0.7%) | 6,755 (0.6%) | 6,734 (0.6%) |
| Malignant neoplasms, colorectal | 30,087 (1.9%) | 114,540 (2.2%) | 21,991 (2.0%) | 21,684 (1.9%) |
| Malignant neoplasms, pancreas | 1,416 (0.1%) | 6,911 (0.1%) | 1,097 (0.1%) | 1,143 (0.1%) |
| Malignant neoplasms, prostate | 66,105 (4.2%) | 195,308 (3.8%) | 45,450 (4.1%) | 45,621 (4.1%) |
| Malignant neoplasms, stomach | 2,471 (0.2%) | 11,691 (0.2%) | 1,884 (0.2%) | 1,898 (0.2%) |
| Acute leukemia | 1,996 (0.1%) | 9,643 (0.2%) | 1,531 (0.1%) | 1,538 (0.1%) |
| Malignant neoplasms, bladder | 22,777 (1.5%) | 68,390 (1.3%) | 15,719 (1.4%) | 15,525 (1.4%) |
| Chemotherapy | 39,684 (2.5%) | 162,515 (3.2%) | 29,925 (2.7%) | 30,269 (2.7%) |
| Antineoplastic use | 44,299 (2.8%) | 187,477 (3.7%) | 33,158 (3.0%) | 33,227 (3.0%) |
| Immunodeficiency/immunosuppression |  |  |  |  |
| Disorders of the immune system | 8,724 (0.6%) | 32,733 (0.6%) | 6,309 (0.6%) | 6,351 (0.6%) |
| Transplant recipient | 6,444 (0.4%) | 11,122 (0.2%) | 3,898 (0.3%) | 3,821 (0.3%) |
| Corticosteroid use | 71,204 (4.5%) | 284,318 (5.6%) | 52,684 (4.7%) | 53,043 (4.7%) |
| Other immunosuppressant use | 10,589 (0.7%) | 45,982 (0.9%) | 7,890 (0.7%) | 7,830 (0.7%) |
| Renal disease |  |  |  |  |
| Chronic renal failure | 66,433 (4.2%) | 103,870 (2.0%) | 39,851 (3.6%) | 39,304 (3.5%) |
| Acute renal failure | 24,813 (1.6%) | 45,887 (0.9%) | 15,267 (1.4%) | 15,206 (1.4%) |
| Nephritis, nephrosis | 10,863 (0.7%) | 17,398 (0.3%) | 6,516 (0.6%) | 6,339 (0.6%) |
| Renal disorders, other | 32,087 (2.1%) | 66,414 (1.3%) | 20,620 (1.8%) | 20,597 (1.8%) |
| Blood disorders |  |  |  |  |
| Hemolytic anemia | 4,567 (0.3%) | 15,096 (0.3%) | 3,217 (0.3%) | 3,241 (0.3%) |
| Iron deficiency, other deficiency anemias | 197,107 (12.6%) | 611,798 (12.0%) | 133,328 (11.9%) | 130,587 (11.7%) |
| Aplastic anemia | 4,967 (0.3%) | 17,374 (0.3%) | 3,514 (0.3%) | 3,441 (0.3%) |
| Conditions associated with increased risk of aspiration |  |  |  |  |
| Dementia and delirium | 68,381 (4.4%) | 333,229 (6.5%) | 49,574 (4.4%) | 48,216 (4.3%) |
| Multiple sclerosis | 2,147 (0.1%) | 9,851 (0.2%) | 1,605 (0.1%) | 1,543 (0.1%) |
| Parkinson's disease | 18,305 (1.2%) | 88,691 (1.7%) | 13,702 (1.2%) | 13,586 (1.2%) |
| Cerebrovascular disease | 193,928 (12.4%) | 441,028 (8.6%) | 124,617 (11.1%) | 123,259 (11.0%) |
| Aspiration history | 37,178 (2.4%) | 138,022 (2.7%) | 26,842 (2.4%) | 26,831 (2.4%) |
| Alcoholism | 44,968 (2.9%) | 174,146 (3.4%) | 31,869 (2.8%) | 31,818 (2.8%) |
|  |  |  |  |  |
| **Other comorbidities** |  |  |  |  |
| Trauma | 56,228 (3.6%) | 220,339 (4.3%) | 41,474 (3.7%) | 41,627 (3.7%) |
| Chronic liver disease | 5,595 (0.4%) | 35,037 (0.7%) | 4,427 (0.4%) | 4,424 (0.4%) |
| Muscle disease | 134,611 (8.6%) | 424,568 (8.3%) | 96,596 (8.6%) | 98,398 (8.8%) |
| Rheumatological disease | 107,668 (6.9%) | 358,198 (7.0%) | 76,683 (6.8%) | 77,038 (6.9%) |
|  |  |  |  |  |
| **Cardiovascular medications** |  |  |  |  |
| Hypolipidemic agents (non-statin) | 66,564 (4.3%) | 163,085 (3.2%) | 49,930 (4.5%) | 56,072 (5.0%) |
| ACE inhibitors | 725,140 (46.3%) | 1,337,419 (26.2%) | 465,094 (41.5%) | 468,261 (41.8%) |
| Angiotensin receptor blockers | 199,237 (12.7%) | 280,238 (5.5%) | 125,643 (11.2%) | 125,844 (11.2%) |
| β blockers | 590,862 (37.8%) | 938,133 (18.4%) | 363,674 (32.5%) | 361,754 (32.3%) |
| Oral anticoagulants | 143,702 (9.2%) | 355,418 (7.0%) | 97,488 (8.7%) | 99,810 (8.9%) |
| Calcium channel blockers | 576,561 (36.8%) | 1,138,358 (22.3%) | 380,522 (34.0%) | 387,180 (34.6%) |
| Nitrates | 355,219 (22.7%) | 525,282 (10.3%) | 210,553 (18.8%) | 204,887 (18.3%) |
| Furosemide | 210,176 (13.4%) | 558,876 (10.9%) | 139,427 (12.4%) | 137,768 (12.3%) |
| Loop diuretics | 210,387 (13.4%) | 559,479 (10.9%) | 139,571 (12.5%) | 137,929 (12.3%) |
| Non-loop diuretics | 443,873 (28.4%) | 1,188,753 (23.3%) | 310,479 (27.7%) | 314,946 (28.1%) |
| Other antihypertensive agents | 94,970 (6.1%) | 252,192 (4.9%) | 65,344 (5.8%) | 66,861 (6.0%) |
| Antiplatelet agents (non-aspirin) | 91,887 (5.9%) | 78,525 (1.5%) | 45,654 (4.1%) | 40,677 (3.6%) |
|  |  |  |  |  |
| **Other medications** |  |  |  |  |
| Bisphosphonates | 217,624 (13.9%) | 592,062 (11.6%) | 158,424 (14.1%) | 160,497 (14.3%) |
| Estrogen replacement therapy | 117,088 (7.5%) | 492,414 (9.6%) | 92,347 (8.2%) | 95,497 (8.5%) |
| Thyroid-active drugs | 235,244 (15.0%) | 684,514 (13.4%) | 168,227 (15.0%) | 170,552 (15.2%) |
| Antipsychotics | 34,140 (2.2%) | 182,567 (3.6%) | 25,502 (2.3%) | 25,139 (2.2%) |
| Antidepressants | 247,386 (15.8%) | 751,688 (14.7%) | 174,873 (15.6%) | 175,956 (15.7%) |
| Sedative-hypnotics | 376,476 (24.1%) | 1,214,132 (23.7%) | 269,427 (24.0%) | 271,889 (24.3%) |
| Cholinesterase inhibitors | 24,220 (1.5%) | 73,173 (1.4%) | 17,298 (1.5%) | 17,804 (1.6%) |
| Parkinson drugs | 15,674 (1.0%) | 74,481 (1.5%) | 11,611 (1.0%) | 11,498 (1.0%) |
| COPD drugs | 261,184 (16.7%) | 826,818 (16.2%) | 184,942 (16.5%) | 185,437 (16.6%) |

AMI = acute myocardial infarction. COPD = chronic obstructive pulmonary disease. ACE = Angiotensin-converting enzyme
